# Retrospective analysis of characteristics and transfer times of helicopter interhospital transfer of stroke patients: balancing air and ground transport efficiency

**DOI:** 10.1186/s13049-025-01506-z

**Published:** 2025-11-15

**Authors:** Dominik Johannes Hoechter, Clemens Rieder, Leopold Kies, Florian Reifferscheid, Jörg Braun, Stephan Prückner, Suzette Heck, Andreas Bayer

**Affiliations:** 1 Department of Anaesthesiology, LMU University Hospital Ludwig-Maximilians-Universität München, Marchioninistr. 15, 81377 Munich, Germany; 2 German Air Rescue Service Association “DRF Luftrettung”, air base Munich, Elisabeth-Stöber-Straße 71, D-81377 Munich, Germany; 3 Scientific research department, German Air Rescue Service Association “DRF Luftrettung”, Rita-Maiburg-Straße 2, D-70794 Filderstadt, Germany; 4 Department of Anesthesiology and Intensive Care Medicine, University Hospital Schleswig-Holstein, Campus Kiel, Kiel, Germany; 5 Institut für Notfallmedizin und Medizinmanagement (INM), Klinikum der Universität München, LMU München, Schillerstr. 53, 80336 München, Germany; 6 Department of Neurology, LMU University Hospital, LMU Munich, Marchioninistrasse 15, 81377 Munich, Germany

**Keywords:** Interhospital transport, Interfacility transport, HEMS, Air-based transport, Ground transport, Stroke, Dispatch

## Abstract

**Background:**

Ongoing changes in the hospital landscape, combined with evolving patient transfer modalities, are reshaping acute care pathways. This study analyzed the medical needs of transferred stroke patients and assessed the potential time advantage of helicopter transport in this shifting context.

**Methods:**

A retrospective analysis of all interhospital helicopter transfers for patients with suspected or confirmed stroke conducted by one HEMS service between 2012 and 2020 was performed. Patient characteristics, medical interventions, and transfer times were extracted from the operational database. Helicopter transfer times were compared to calculated ambulance driving times, which were estimated using Google Maps data adjusted by a correction factor accounting for driving with lights and sirens.

**Results:**

Out of 179,000 total missions, 7,528 were interhospital stroke transfers. Among these, 6,273 patients (83.3%) were classified as NACA 3 or 4, and 1,154 (13.3%) required mechanical ventilation. 2,227 patients (29.6%) received no medical intervention during transport. Complete transfer times and distances were available for 5,243 missions. The median total flight time—from alert to landing at the receiving hospital—was 49 min (IQR 37–63), compared to a median calculated driving time of 54 min (IQR 44–67). In 1,873 cases (62.8%), the total flight time was shorter than the estimated driving time, with a median time difference of − 3.4 min (IQR − 14.1 to + 7.3).

**Conclusions:**

Since many stroke patients required no interventions during transport, careful patient selection could help reserve specialized transfer resources for the most critical cases. While air transport provided a time advantage for patients in many cases, it was not always the fastest mode of transfer. Future design of emergency medical resources and dispatch strategies should be guided by the estimated time of patient arrival at the accepting hospitals.

**Supplementary Information:**

The online version contains supplementary material available at 10.1186/s13049-025-01506-z.

## Background

Stroke is a leading cause of morbidity and mortality worldwide [1–3]. In Germany, it ranks as the third most common cause of death and is one of the leading reasons for acquired disability in adults [4]. Rapid diagnosis and treatment are crucial to improving neurological outcomes. Today, computed tomography (CT) as well as intravenous thrombolysis with recombinant tissue-type plasminogen activator (rt-PA) are widely available and correspond to guideline-based therapy. Endovascular therapy has proven beneficial but remains limited to specialized stroke centers providing neurointerventional treatment [5, 6]. Thus, patients qualifying for endovascular treatment are frequently transferred from community hospitals to tertiary-care centers for advanced management. Helicopter transport is an established method for these time-critical transfers [7]. Approximately 8% of all helicopter emergency medical service (HEMS) missions in Germany are dedicated to secondary transfers of stroke patients, making stroke one of the top three diagnoses for interhospital transfers [5].

In the coming years, hospital structures and patient transfer systems are expected to undergo substantial changes, driven by the centralization of specialized care and advancements in transportation technology. These developments may include not only reorganization of existing ground and air transport capacities but also the introduction of novel concepts such as electric vertical take-off and landing (eVTOL) aircraft and autonomous drones [8–10]. As these new systems are designed and implemented, robust benchmark data from current HEMS operations are needed to inform cost–benefit considerations and guide the efficient use of future resources.

This study aims to analyze the medical needs of stroke patients transported by helicopter and to quantify the potential time advantage of helicopter transport compared to ground ambulance transfer.

## Methods

A retrospective analysis of the German air rescue service DRF Luftrettung (DRF Luftrettung gAG, Filderstadt, Germany) database was conducted. The database collects all operational data of the 29 DRF helicopters in Germany. The helicopters are part of both emergency medical services and the interhospital retrieval network. The medical crew on the helicopters consists of a pre-hospital emergency physician (mostly specialists in anesthesiology, surgery, or internal medicine) and a HEMS-TC (helicopter emergency medical service technical crew member) qualified as a paramedic. Each mission is documented in a standardized online database (HEMSDER-Database, Convexis, Germany). The collected data included flight times as well as medical details on the patient and care.

This retrospective study included all missions of DRF helicopters with a patient transported from 2012 to 2020. Interhospital retrieval missions due to the primary diagnosis “stroke” were identified. For these missions, the following times were extracted: the time from alert to landing at the referring hospital, the flight time from the referring to the accepting hospital, and whether an intermediate transport to or from the helicopter landing site was necessary. Patient characteristics, including age, sex, and diagnosis, were retrieved, as well as information about medical treatment during the patient transport (intubated/ventilated, need for vasopressors, use of syringe infusion pumps (SIPs).

In Germany, interhospital patient transfers can also be conducted by ground ambulance. In selected cases, so-called “retrieval physicians” – specialized emergency or intensive care physicians – may accompany the patient in the ambulance, ensuring a standard of medical care similar to HEMS during transfer.

For a comparison of flight times against presumed driving times of ground ambulances, estimates of ambulance driving times were determined using the Google Maps API (https://developers.google.com/maps). To account for the potential use of emergency lights and sirens, driving times were adjusted by a factor of 0.74 [11]. For total driving times, a default response time of 8.7 min was added, representing the ambulance’s response time to the referring hospital [12]. Estimated driving times were compared to the sum of helicopter response times (from alert to landing at the referring hospital) and transportation time (flight time from the referring to the accepting hospital). Flight times included both engine warm-up and cool-down times. The duration of patient handovers was omitted, based on the assumption that it did not vary between systems.

For the descriptive analysis of numerical variables, the median and interquartile range (IQR) - or, in case of normal distribution, the mean and standard deviation (SD) - were reported. For categorical and dichotomous variables, absolute frequencies and proportions (percentages) were calculated. A *p*-value of ≤ 0.05 was considered statistically significant. Since transfer times were not normally distributed (as determined by the Shapiro-Wilk test), a Wilcoxon signed-rank test was applied to compare the transfer modes. All analyses were performed using R (version 4.5.1), Microsoft^®^ Excel for Mac (version 16.92, 24120731), XLSTAT Cloud (version 5.0.1, Lumivero, Denver, CO, USA), and numiqo Team (2025). numiqo: Online Statistics Calculator. DATAtab e.U. Graz, Austria. URL https://numiqo.de.

## Results

Between 2012 and 2020, DRF helicopters realized 179,000 patient transports. 84,349 (47.1%) were interhospital retrieval missions (secondary missions). In 7,528 (8.9% of all secondary retrieval missions) cases, stroke was documented as the primary diagnosis. 1,590 missions (21.1%) took place during nighttime, and 5,833 (77.5%) took place during daytime, respectively. In 199 cases (2.6%), the patient was transported by ground ambulance accompanied by the HEMS medical crew. In 3,241 of the 7,329 air transports (44.2%), an intermediate transport by ambulance was required between the hospital and the landing site (Table 1).

**Table 1:** Overview of HEMS mission characteristics and calculated ground ambulance driving time. The number and key characteristics of missions performed. Key time metrics are provided as medians with interquartile ranges. Percentages are related to the total of 7,528 interhospital stroke transfers. *The calculated ground ambulance driving time includes a default response time of 8.7 min

all transports [*n*]	179,000	
interhospital transfer missions [n]	84,349	
interhospital stroke transfer [n]	7,528	
nighttime missions [n]	1,590	21.1%
dispatch urgency [n]		
high urgency [n]	555	7.3%
regular urgency [n]	6,973	92.6%
response time [min]	26	(18–36)
transfer time [min]	21	(16–28)
calculated driving time* [min]	54	(44–67)

The median response time (alert to landing at the referring hospital) was 26 min (IQR 18–36 min), and the median flight time from the referring to the accepting hospital was 21 min (IQR 16–28 min). Total flight time from alert to landing at the receiving hospital was a median of 49 min (IQR 37–63 min). The median aerial (straight-line) distance between hospitals was 53 km (IQR 37–67 km).

**Fig. 1:**
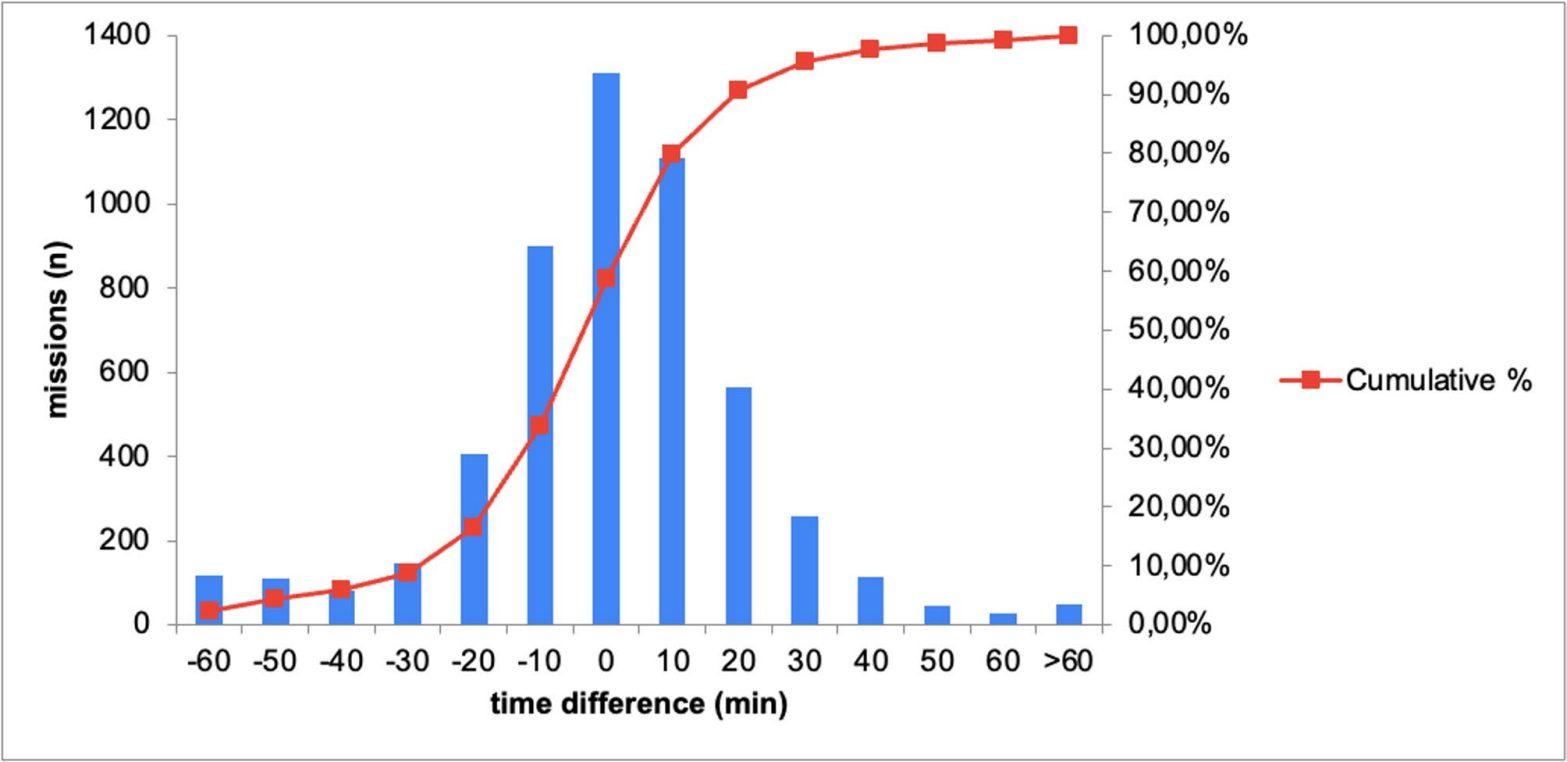
Histogram illustrating the time differences between helicopter transfers and calculated ground ambulance driving times. Negative values indicate faster arrival by helicopter, while positive values reflect faster arrival by ground transport. The number below the bar indicates the upper limit of the interval

Complete transfer times and driving distances were available for 5,243 missions. For each of these missions, the corresponding ambulance driving time was estimated. The median estimated patient transfer time was 45 min (IQR, 36–58 min), and the estimated total driving time was 54 min (IQR, 44–67 min). ​​As assessed by the paired Wilcoxon signed-rank test, total flight time and driving time differed significantly (*p* < 0.001). The duration of ground-based transportation was shorter in 2,169 cases (41.4%). In 3,074 cases (58.6%), total flight time was shorter than the calculated driving time. The median time difference was − 3.4 min (IQR − 14.1 to + 7.3 min) (Fig. 1).

Demographics, severity of illness or injury expressed via the National Advisory Committee for Aeronautics (NACA) and vital signs including Glasgow Coma Score (GCS), peripheral oxygen saturation (SpO_2_), heart rate and systolic blood pressure are depicted in Table 2 (Table 2). 3,944 (52.4%) of the patients were male. Patients had a median age of 72 years (IQR 60–80 years) and were severely afflicted, with 6,291 (83.5%) scored NACA 4 or higher. Among 4,730 (62.8%) patients who initially presented with a GCS of higher than twelve, 4,177 (55.8%) did not decrease in GCS during transport. All but a few (7,277 (96.7%)) presented with SpO2 values greater than 90% throughout the transport. Heart rate remained stable in the range of 50 to 120 beats per minute in 6,938 (92.1%) patients. In 5714 (76,7%) patients, systolic blood pressure was between 120 and 179 mmHg (Table 2).

**Table 2:** Demographic and baseline clinical characteristics at the start of the transfer. Categorical variables are reported as absolute numbers and percentages. Age is provided as the median with an interquartile range. The percentages were calculated based on the total population of 7,528 patients; the number of valid observations for each variable is indicated in parentheses

all stroke interhospital transfers	*n* = 7,528	
gender (*n* = 7,351)		
female	3,407	45,3%
male	3,944	52,3%
age [years] (*n* = 7,528)	72	60–80
NACA score (*n* = 7,527)		
NACA 3	1,233	16.4%
NACA 4	2,915	38.7%
NACA 5	3,358	44.6%
NACA 6/7	18	0.2%
GCS (*n* = 7,526)		
15	3,234	43.0%
>12	4,730	62.8%
<9	1,493	19.8%
SpO_2_ in percent (*n* = 7,469)		
95–100	5,835	77.5%
90–94	1,459	19.4%
heart rate in beats per minute (*n* = 7,455)		
60–99	5,756	76.5%
<60	654	8.7%
≥100	1,032	13.7%
systolic blood pressure in mmHg (*n* = 7,460)		
120–179	5,714	75.9%
<120	749	9.9%
≥180	985	13.1%

1,154 (13.3%) patients were mechanically (invasively) ventilated. 27 (0.4%) received non-invasive ventilation, i.e., continuous positive airway pressure ventilation (CPAP) or CPAP with augmented spontaneous breathing (CPAP/ASB). 3,371 (44.8%) patients received oxygen supplementation ranging from 1 to 6 L per minute via nasal cannula or open oxygen mask. 3,189 (42.36%) patients did not receive any respiratory support (Table 3). During the retrieval, opioids (fentanyl, morphine, sufentanil) were administered to 239 (3.17%) patients, narcotics (propofol, ketamine, midazolam, etomidate) to 638 (8.48%) patients, and muscle relaxants to 186 (2.47%) patients. 365 (4.85%) patients were treated with vasopressors and inotropic substances such as noradrenaline, dobutamine, and theodrenaline-cafedrine, while 693 (9.2%) patients received antihypertensive treatment (urapidil). In 1079 (14.3%) cases, antiemetics were administered. In 1,635 (21.7%) transports, syringe infusion pumps were used to administer one or more medications continuously.

**Table 3:** Listing the interventions and medications administered. Categorical variables are reported as absolute numbers and percentages. The percentages were calculated based on the total population of 7,528 patients

all stroke interhospital transfers	*n* = 7,528	
oxygen supplementation	3,371	44.8%
non-invasive ventilation	27	0.4%
invasive ventilation	1,154	13.3%
crystalloid infusion	5,866	77.9%
vasoactive medication	1,058	14.1%
vasopressors/inotropes	365	4.8%
antihypertensive medication	693	9.2%
opioids	1,035	13.7%
sedatives/narcotics	1,468	19.5%
muscle relaxants	341	4.5%
antiemetics	1,079	14.3%
no medical intervention performed	2,227	29.6%

2,227 (29.6%) patients received no intervention at all, 4,034 (53.6%) received no intervention but oxygen therapy, and 4,838 (64.3%) patients received no intervention but supplemental oxygen or antiemetics.

## Discussion

This study described the medical characteristics of stroke patients transferred to comprehensive stroke centers offering interventional treatment after initial diagnosis and treatment in a primary hospital.

Most of the patients were critically ill, with 83.5% labeled as NACA 4 or higher. A substantial proportion required relevant medical support during transport: 13% were invasively ventilated during the transfer, vasoactive or antihypertensive agents were administered in over 14% and 9% of cases, respectively. Nonetheless, 30% of patients received no intervention at all, and more than half were treated with oxygen therapy only. Median mission response time was 26 min, with a median transportation time between hospitals of 21 min. While total flight time from alert to arrival at the receiving hospital was slightly longer than the estimated driving time in many cases, air transport resulted in earlier arrival at the destination hospital in over half of the missions, underlining its logistical and clinical relevance in time-sensitive stroke care.

**Fig. 2:**
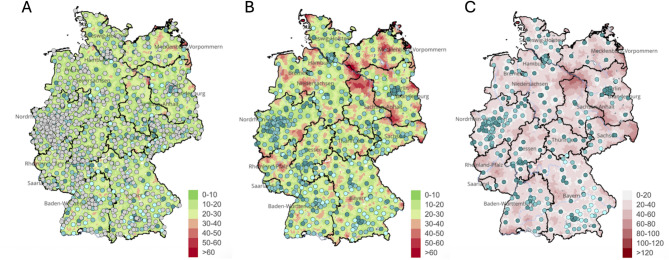
Map of Germany showing the travel time in minutes to the nearest hospital of the respective level of care: (**A**) Primary care hospitals offering basic diagnostics and intravenous thrombolysis; (**B**) Hospitals with specialized stroke units; (**C**) Comprehensive Stroke Centers (CSC) providing endovascular treatment [20]. (Reprinted with the kind permission of the Science Media Center)

Stroke remains one of the leading causes of morbidity and mortality worldwide. In ischemic stroke, timely initiation of intravenous thrombolysis and mechanical thrombectomy is crucial as faster treatment consistently improves outcomes [5, 13, 14, 15–19]. In recent years, several prehospital and interhospital emergency care models have been developed to expedite stroke treatment: While the “**mothership**” approach facilitates immediate thrombectomy by directing patients to comprehensive stroke centers, it also may contribute to capacity strains at comprehensive centers and can prolong the prehospital times especially in rural regions - in Germany from 9.5 min to approximately 18 min (Fig. 2) [20]. In the “**drip-and-drive**” concept, mobile stroke units (MSUs) equipped with CT imaging and thrombolytic therapy are directly deployed to the scene, significantly reducing onset-to-treatment times in pilot projects [21]. However, high costs and logistical demands limit its availability. Adaptations include CT-equipped helicopters or rapid blood tests to differentiate ischemic from hemorrhagic stroke [22–25]. A related concept involves transferring the interventionalist to a primary stroke center, although this remains constrained by the availability of infrastructure [25, 26]. Currently, the most widespread model is the “**drip-and-ship**” model, in which patients receive initial treatment at a primary stroke center before being transferred to a neurovascular hub. This strategy introduces secondary transfer delays of 40–130 min [26–30]. Notably, this delay is not only caused by the transport itself but also arises from medical decision-making and the logistics of arranging the transfer [26, 27]. Such delays are clinically relevant, as shorter transfer times correlate with higher thrombectomy rates and improved outcomes [28, 29, 31]. Importantly, Ribo et al. showed that each 30-minute reduction from imaging to reperfusion increases the likelihood of a favorable outcome by 28% [32].

Overall, treatment delays are likely multifactorial in nature. Earlier symptom recognition, timely activation of emergency medical services, and streamlined in-hospital workflows may provide equal or even greater time savings than faster interhospital transfer alone. Nevertheless, it is crucial to identify the most expeditious means of transport from referring hospitals to comprehensive stroke centers. In the present study, helicopter transfer was superior to ground transport in 58.6% of missions. Numerous stroke networks have published retrospective and descriptive data on both ground and air transport of stroke patients, with many studies concluding that air transport is generally faster [7, 28–30, 33–35]. However, these analyses often lacked direct comparisons of transport times, as specific location data were either not recorded or not available for both transport modalities. Moreover, most studies reported only patient transfer times without accounting for response times. The latter might offset the time savings of a speedy helicopter flight, especially at nighttime, as a reduced number of operational helicopters in Germany—17 compared to 84 during the daytime—leads to greater inbound flight distances. This effect was also observed in the present study: the median patient transport time in HEMS was 21 min, but the median total transfer time added up to a median of 49 min.

As far as the published literature reported, dispatch and arrival times varied considerably between study regions: dispatch times of 5 ± 6 min and 17 ± 8 min for ground ambulances and helicopters, respectively, as well as ambulance arrival times at the referring hospital of 25 ± 12 min, were reported in one paper [36]. Compared to mean dispatch times of 2.5 to 3.8 min and mean ambulance arrival times of 8.7 to 13.1 min in Germany, this highlights the geographical and logistical characteristics of the individual study settings and the limited generalizability of these data [12, 37].

For the present study, we aimed to rely on evidence-based data: The decision to use 8.7 min as the estimated ambulance response time was guided by evidence from a nationwide study [12]. Likewise, the applied speed correction factor of 0.74 was derived from an empirical analysis of 4,479 documented ambulance journeys in Germany [11]. In similar projects, ambulance travel times were estimated using navigational software, with driving speeds either derived from a set of only 30 recorded ambulance journeys or assumed where data were lacking [37]. Although even more advanced methods for estimating ambulance driving times, such as Bayesian data augmentation, are available, we chose not to apply them, as they were deemed unnecessarily complex for the scope and purpose of this analysis [38, 39].

Transfer times from the helicopter landing site or ambulance bay to the emergency department or angiography laboratory, as well as patient handover times, were not considered in this analysis. To the best of our knowledge, no published data exist on handover duration during interhospital transfer, nor on differences between transport modalities. Given the nationwide, ten-year scope of this dataset, variations in intrahospital distances could not be assessed uniformly. Even though there is an increasing adoption of rooftop helipads, in-hospital transfers can still consume valuable minutes. As these unaccounted intervals may further prolong total transfer time, it is worth noting that in 1,310 missions (25%), the helicopter’s time advantage was less than ten minutes. Conversely, in 1,100 missions (21.2%) ground transport was no more than ten minutes faster, illustrating that time differences between transport modes are often marginal.

Regarding helicopter transfers that require an intermediate ground transport between the landing site and the hospital, these missions were included in the primary analysis, as previous data from the same dataset indicated that this adds only a few minutes to overall transport time [40]. To address this bias, we conducted a secondary analysis, excluding all missions requiring intermediate ambulance transports (*n* = 2,984) and transfers performed by ground ambulance accompanied by HEMS medical crew which yielded comparable results to the primary analysis with air based transfers significantly faster, with a median time difference of -5.0 min (IQR − 15.8 to + 5.1); these results are provided in an additional file (Additional File 1).

Nevertheless, in this study, helicopter transfer was estimated to be slower than ground transport in over 40% of cases. This phenomenon, where helicopter dispatch—initially selected as the presumed fastest option—ultimately leads to delays, has also been observed in other emergency service contexts [41]. In such situations, the benefit of reducing the time a patient spends in the transport vehicle (ambulance or helicopter) must be carefully weighed against the advantage of earlier initiation of definitive care at the receiving facility. In the present study, the median patient transport time in HEMS was 21 min and thus considerably shorter than the calculated patient transport time by ground services of 45 min. However, despite a slightly shorter total transfer time in the HEMS group (median 49 vs. 54 min), helicopter transfer was still slower than ground transport in a substantial proportion of cases.

To ensure the fastest possible transport, dispatchers should continuously reassess evolving situations, potentially supported by dispatch software that not only estimates time to scene arrival but also continuously predicts the estimated time of patient arrival (ETPA) at the destination hospital. While such systems are believed to be in use in certain regions, we found no published evidence confirming their implementation.

From a health-economic perspective, helicopter transfers are associated with considerably higher operational costs than ground transport, mainly due to maintenance, staffing, and standby readiness requirements. However, these costs may be offset when time savings translate into improved outcomes, particularly in time-sensitive conditions such as trauma or stroke [42]. Cost-effectiveness depends not only on the quality of medical care but also strongly on the magnitude of the time advantage achieved, underscoring the importance of targeted and evidence-based HEMS utilization.

In the future, electric Vertical Take-Off and Landing (eVTOL) may provide an alternative means of air transfer with lower acquisition and operating costs yet similar speed advantages. While empirical data are lacking, manufacturers report projected cruising speeds of 180–250 km/h for eVTOLs, which are broadly comparable to the operational speeds of 230–250 km/h documented for Airbus H135 and H145 helicopters, commonly used in German HEMS [43–47]. Payload and range limitations of current eVTOL prototypes make routine use appear distant, but could be overcome by establishing a denser network of eVTOL bases.

In addition to considerations of transport times, the medical implications of air transfer merit discussion. Air medical transport of stroke patients—including those who have received intravenous rt-PA—is safe [34, 48]. While some authors concluded that empirical studies showed a mixed picture of using HEMS for secondary transfer of stroke patients and ground transportation not being associated with a greater risk of complications or deterioration en route, others reported benefits of helicopter transport extending beyond mere time savings, particularly in patients who achieved successful mechanical thrombectomy [29, 34]. One possible explanation proposed by the authors is that the low-frequency vibrations characteristic of helicopter flights—typically around 50 Hz—may exert thrombolytic effects, potentially enhancing the efficacy of intravenous thrombolysis and facilitating reperfusion [49]. Alternatively, higher-quality ancillary care provided by experienced and specialized personnel on board was suggested as a contributing factor to the improved outcomes. In most systems, HEMS crews consist of flight nurses with critical care training or emergency physicians with backgrounds in intensive care or anesthesia. Evaluating the added value of flight physicians in HEMS, Rhee et al. found that they made a unique and essential contribution to patient care in 22% of missions, primarily through clinical judgment in diagnosis, initiation of critical treatments, and determination of the appropriate destination hospital [50].

Due to cost constraints, limited availability of flight physicians and nurses, and notably the restricted payload capacity of eVTOL aircraft, it is essential to carefully consider the personnel requirements and qualifications necessary for transporting patients safely and effectively.

In the present study, the majority of patients were classified as being in a potentially life-threatening condition, with 83.3% assigned to NACA categories 4 or 5, and 13.3% requiring mechanical ventilation. However, 1,970 (26.2%) patients presented in stable condition during the transport, and 2,227 (29.6%) patients received no medical intervention during the transport, pointing to the inherent subjectivity of the NACA score [51].

A total of 3,880 patients (51.5%) received antiemetics and/or supplemental oxygen. Antiemetics are often administered prophylactically in (air-based) interhospital transfer prior to take-off to prevent nausea; in addition, dimenhydrinate provides a mildly calming side effect. Excluding the administration of antiemetics and low-dose oxygen supplementation, 4,838 patients (64.3%) required no significant medical intervention that would necessitate the presence of a specialized emergency physician.

These findings suggest that the majority of interhospital transfers in this cohort could be safely managed by paramedic-only crews, without the routine need for specialized medical personnel. This observation may inform future considerations regarding crew composition for eVTOL aircraft and helicopters in interfacility transport, as well as ongoing discussions about the necessity of retrieval physicians in ground-based retrieval services—particularly in light of advances in real-time telemedical support. However, prudent selection of patients and suitable transport modalities at the referring hospital will become essential—a process that may be aided by telemedical support in the future.

This study has several limitations inherent to its retrospective design, including potential documentation bias and the inability to control for unmeasured confounders. Ground transport times were estimated using average driving times, which did not account for real-time factors such as road or weather conditions. One of the most significant limitations is the lack of detailed clinical data regarding the neurological status of the patients: NIHSS scores were not available and could not be deduced from the available medical records. Furthermore, no information was available regarding the acute treatment provided at the primary stroke centers, subsequent interventions at the comprehensive stroke centers, or functional outcomes. Finally, an economic evaluation could not be included within the scope of this study.

## Conclusion

Our study provides a detailed analysis of air-based interhospital transfers of stroke patients. Since a significant proportion of patients did not require medical interventions during transport, prudent patient selection could help focus the use of retrieval physicians and specialized transport teams on the most critically ill patients. While air transport can offer a notable time advantage over ground transfer, it is not consistently the fastest option. Future design of emergency medical resources and dispatch strategies should be guided by the estimated time of patient arrival at the comprehensive stroke center.

## Supplementary Information

Below is the link to the electronic supplementary material.

Supplementary Material 1

## Data Availability

The data supporting the findings of this study are available from the German Air Rescue Service Association “DRF Luftrettung” and can be shared upon reasonable request. However, open access is restricted due to licensing agreements.

## Abbreviations

CPAP: Continuous positive airway pressure ventilation
CPAP/ASB: Continuous positive airway pressure ventilation - augmented spontaneous breathing
CSC: Comprehensive stroke center
CT: Computed tomography
eVTOL: Electric Vertical Take-Off and Landing
GCS: Glasgow Coma Scale
HEMS: Helicopter emergency medical service
HEMS-TC: Helicopter emergency medical service technical crew member
IQR: Interquartile range
MSU: Mobile stroke unit
NACA: National Advisory Committee for Aeronautics
NIHSS: National Institute of Stroke Scale
PSC: Primary stroke center
rt-PA: Recombinant tissue-type plasminogen activator
SpO2: Peripheral oxygen saturation
