# Supplementary material for: Retrospective analysis of characteristics and transfer times of helicopter interhospital transfer of stroke patients: balancing air and ground transport efficiency
Source: Scand J Trauma Resusc Emerg Med. 2025 Nov 15;33:184. doi: 10.1186/s13049-025-01506-z (PMC12619459; doi:10.1186/s13049-025-01506-z)
Supplement: Supplementary file 1 — Supplementary Material 1 [file 13049_2025_1506_MOESM1_ESM.docx]

Additional file 1:

Secondary analysis: comparison of HEMS and ground ambulance transfers of stroke patients omitting ground ambulance accompanied by the HEMS medical crew as well as air transport that required an intermediate transport by ambulance between the hospital and the landing site


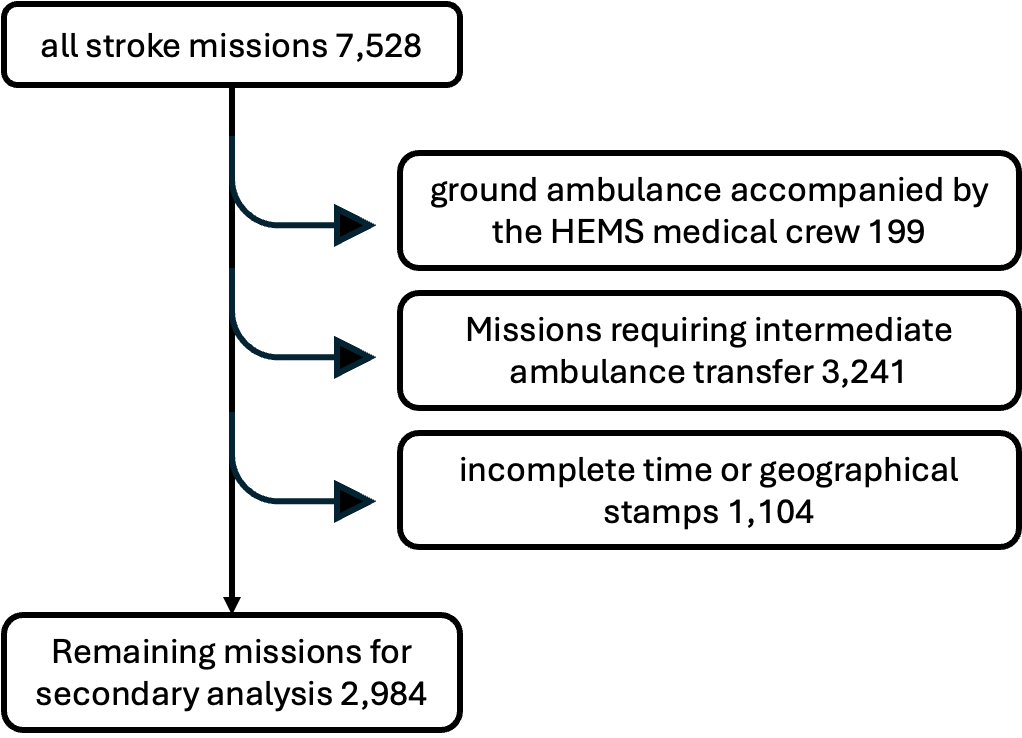


Figure AF1: Flow diagram of the exclusion/ inclusion of transfers for the secondary analysis


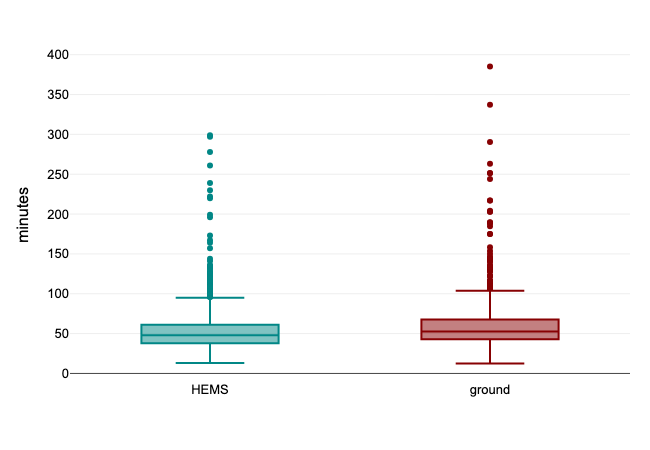


Figure AF2: Box plot of transfer times of the secondary analysis: air transport was significantly shorter than ground transport (p>0.001). Median difference was -5.0 minutes (IQR -15.8 to +5.1)


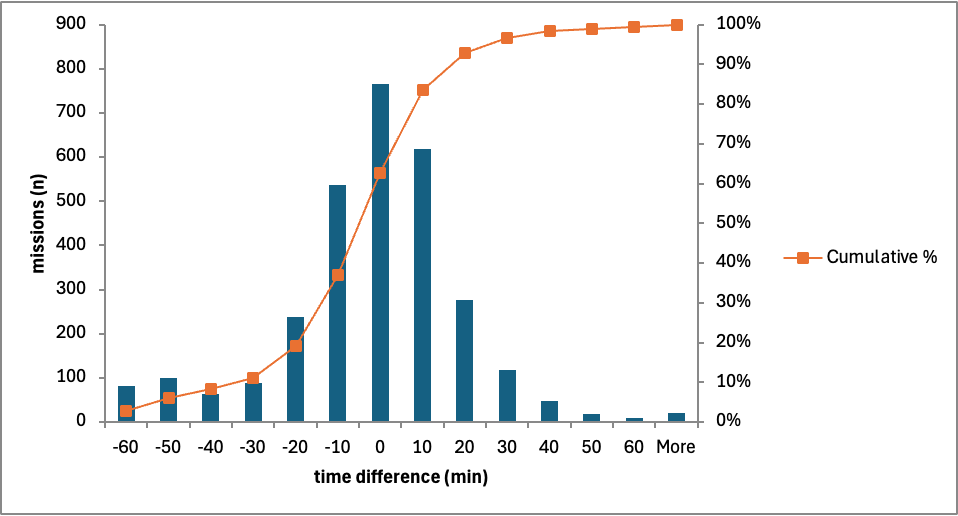


Figure AF3: Histogram of the time difference of air transport versus ground based transfer: In 62.8% of the missions, air transfer was faster, in 25.6% of the missions the air transfer was less then ten minutes faster. In 37.2% of missions, ground based transport was faster.


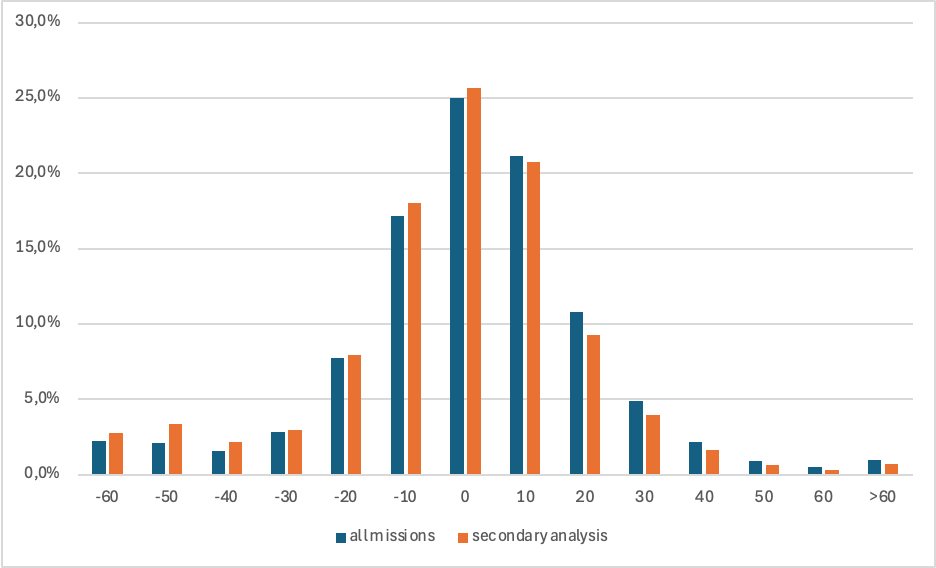


Figure AF4: Histogram of the distribution of time differences of air transport versus ground based transfer for all missions (blue bars) and selected missions (please see above, secondary analysis, orange bars).
